# Methamphetamine Intoxication in Emergency Departments of Hospitals in Iran: Implications for Treatment

**Published:** 2013-12

**Authors:** Zahra Alam Mehrjerdi, Alireza Noroozi

**Affiliations:** 1 Iranian National Center for Addiction Studies, Tehran University of Medical Sciences, Tehran, Iran;; 2 School of Advanced Technologies in Medicine, Iranian National Center for Addiction Studies, Tehran University of Medical Sciences, Tehran, Iran

**Dear Editor,**

Methamphetamine is a potent neurotoxin which can cause dopaminergic degeneration.^1^ In emergency department settings of hospitals, common presenting symptoms relating to Methamphetamine intoxication include chest pain, hypertension, shortness of breath, and tachycardia.^2^ In Iran, Methamphetamine intoxication has recently emerged as a crucial health problem in clinical and therapeutic settings. For example, in a study on 2,325 admitted amphetamine and opioid-intoxicated patients in Aliasghar Hospital in Isfahan, 542 (23.3%) patients reported using amphetamines, while the remaining patients reported co-administration of opioids and amphetamines.^3^ In a study on the prevalence and complications of drug-induced seizures in Baharloo Hospital in Tehran, the capital city of Iran, 143 patients were examined. The study findings showed that Methamphetamine was the most common reason for emerging seizures.^4^ Methamphetamine was also a main reason associated with complications, death, and intoxication.^4^ In another study, Nikkhah et al. examined 4 Methamphetamine-intoxicated patients admitted to the emergency department setting of a hospital in Mashhad. Methamphetamine intoxication resulted in the death of 3 cases.^5^

Managing Methamphetamine intoxication is a treatment priority, but there is a paucity of research on Methamphetamine intoxication in Iran. Several important issues should be considered when the problem of Methamphetamine intoxication is studied. First, there is a dearth of research on the prevalence of Methamphetamine intoxication and its side effects on health. In addition, literature on the prognostic features and clinical manifestations among Iranian patients admitted to the emergency department settings of hospitals is not well-documented and requires research. Second, Iranian patients experiencing Methamphetamine intoxication may present to emergency department settings with life-threatening health problems; such clinical and treatment implications are of great significance and should be considered in the management of Methamphetamine intoxication. Third, emergency medicine specialists should specifically diagnose the signs and symptoms of Methamphetamine intoxication in intoxicated patients because Methamphetamine use could share many of the same toxic clinical effects observed with other stimulants and substances. Therefore, implementing the differential diagnosis of the problem is a medical priority. Fourth, Iranian emergency medicine specialists should note that in the procedures of assessment and diagnosis, clinical observation of toxic signs is a factor even more important than determining the dose of abuse. Emergency room visits associated with Methamphetamine use are more likely to require greater utilization of services than the visits of the average emergency room patients.^2^ Consequently, it is necessary to design and implement specific educational and training courses on the treatment of Methamphetamine intoxication. Fifth, toxic responses to Methamphetamine may include severe cardiovascular and behavioral disturbances, including seizures and stroke.^2^ For serious cases, a supportive care in an emergency department room with an emergency medicine specialist is required. For mild cases, supportive care, regular observation, and consideration of sedation with a benzodiazepine or an antipsychotic medication are the treatment priorities. The role of emergency department settings in response to Methamphetamine intoxication encompasses immediate assessment, diagnosis, and safe management of the symptoms of intoxication, including acute behavioral disturbances and medical complications. 

To sum up, the few targeted efforts aimed at reducing and/or preventing the problem of Methamphetamine intoxication demonstrate that a strategic program of research, prevention, and treatment is required to address Methamphetamine intoxication. Further studies on the effectiveness of the implemented treatment services are required.
